# Non-centrosymmetric Na_3_Nb_4_As_3_O_19_

**DOI:** 10.1107/S1600536812010537

**Published:** 2012-03-17

**Authors:** Saïda Fatma Chérif, Khaled Hizaoui, Mohamed Faouzi Zid, Ahmed Driss

**Affiliations:** a Laboratoire de Matériaux et Cristallochimie, Faculté des Sciences, Université de Tunis El Manar, 2092 El Manar Tunis, Tunisia

## Abstract

A new non-centrosymmetric compound, tris­odium tetra­niobium triarsenic nona­deca­oxide, Na_3_Nb_4_As_3_O_19_, has been synthesized by a solid-state reaction at 1123 K. The structure consists of AsO_4_ tetra­hedra and NbO_6_ octa­hedra sharing corners to form a three-dimensional framework containing two types of tunnels running along the *c* axis, in which the sodium ions are located. Na^+^ cations occupying statistically several sites, respectively, are surrounded by seven, six and four O atoms at distances ranging from 2.08 (1) to 2.88 (4) Å. The title structure is compared with those containing the same groups, *viz.* *M*_2_XO_13_ and *M*_2_ *X*_2_O_17_ (*M* = transition metal, and *X* = As or P).

## Related literature

For physical properties of this class of compound, see: Masquelier *et al.* (1995 ▶); Daidouh *et al.* (1997 ▶, 1998 ▶, 1999 ▶); Sanz *et al.* (1999 ▶, 2001 ▶); Baies *et al.* (2006 ▶); Ravez *et al.* (1972 ▶, 1974 ▶); Torardi *et al.* (1985 ▶); Krol *et al.* (1980 ▶); Blasse *et al.* (1992 ▶). For synthetic details, see: Zid *et al.* (1988 ▶, 1989 ▶); Ben Amor & Zid (2005 ▶, 2006 ▶); Hizaoui *et al.* (1999*a* ▶, *b* ▶); Haddad *et al.* (1988 ▶); Harrison *et al.* (1994 ▶); Chérif *et al.* (2011 ▶). For related structures, see: Guyomard *et al.* (1991 ▶); Serra & Hwu (1992 ▶); Ben Amor & Zid (2006 ▶); Ledain *et al.* (1996 ▶); Leclaire *et al.* (1994 ▶); Amos & Sleight (2001 ▶). For details of the bond-valences method, see: Brown & Altermatt, (1985 ▶).

## Experimental

### 

#### Crystal data

Na_3_Nb_4_As_3_O_19_
*M* *_r_* = 969.37
Orthorhombic, 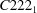

*a* = 13.014 (2) Å
*b* = 24.170 (3) Å
*c* = 5.0880 (9) Å
*V* = 1600.4 (3) Å^3^
*Z* = 4
Mo *K*α radiation
μ = 9.13 mm^−1^
*T* = 298 K
0.35 × 0.25 × 0.16 mm

#### Data collection

Enraf–Nonius CAD-4 diffractometer
Absorption correction: ψ scan (North *et al.*, 1968 ▶) *T*_min_ = 0.083, *T*_max_ = 0.230
2116 measured reflections
1757 independent reflections
1516 reflections with *I* > 2σ(*I*)
*R*_int_ = 0.041
2 standard reflections every 120 min intensity decay: 1%

#### Refinement

*R*[*F* ^2^ > 2σ(*F* ^2^)] = 0.029
*wR*(*F* ^2^) = 0.076
*S* = 1.03
1757 reflections
159 parameters
2 restraints
Δρ_max_ = 0.75 e Å^−3^
Δρ_min_ = −0.91 e Å^−3^
Absolute structure: Flack (1983 ▶), 718 Friedel pairs
Flack parameter: 0.019 (16)

### 

Data collection: *CAD-4 EXPRESS* (Duisenberg, 1992 ▶; Macíček & Yordanov, 1992 ▶); cell refinement: *CAD-4 EXPRESS*; data reduction: *XCAD4* (Harms & Wocadlo, 1995 ▶); program(s) used to solve structure: *SHELXS97* (Sheldrick, 2008 ▶); program(s) used to refine structure: *SHELXL97* (Sheldrick, 2008 ▶); molecular graphics: *DIAMOND* (Brandenburg, 1998 ▶); software used to prepare material for publication: *WinGX* (Farrugia, 1999 ▶).

## Supplementary Material

Crystal structure: contains datablock(s) I, global. DOI: 10.1107/S1600536812010537/ru2026sup1.cif

Structure factors: contains datablock(s) I. DOI: 10.1107/S1600536812010537/ru2026Isup2.hkl

Additional supplementary materials:  crystallographic information; 3D view; checkCIF report

## Figures and Tables

**Fig. 1.**
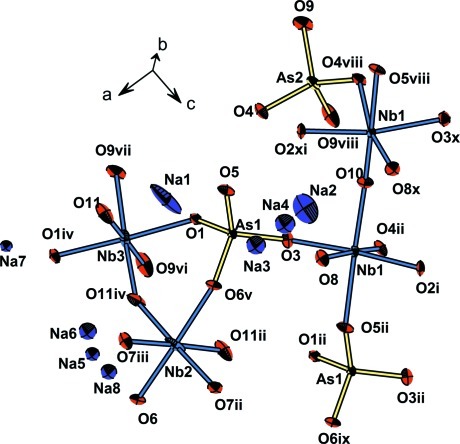
: Unité structurale dans Na3Nb4As3O19. Les éllipsoïdes ont été définis avec 50% de probabilité. [Code de symétrie: (i)-x, -y + 1, z + 1/2; (ii)x, y, z + 1; (iii)-x + 1, -y + 1, z + 1/2; (iv)-x + 1, y, -z + 1/2; (v)-x + 1, y, -z + 3/2; (vi)-x + 1/2, -y + 3/2, z + 1/2; (vii)x + 1/2, -y + 3/2, -z; (viii)-x, y, -z + 1/2; (ix)-x + 1, y, -z + 5/2; (x)-x, y, -z + 3/2; (xi)-x, -y + 1, z - 1/2].

**Fig. 2.**
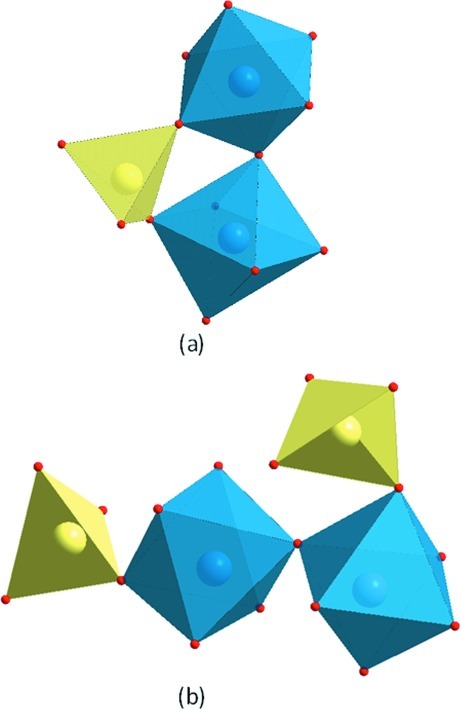
: Représentation: (a) du groupement classique cyclique Nb2AsO13 et (b) de l'unité originale Nb2As2O17.

**Fig. 3.**
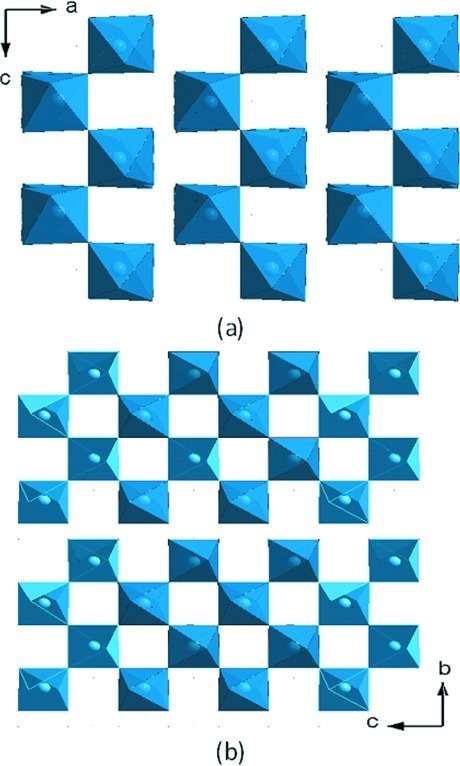
: Représentation: (a) des chaînes ondulées de type [Nb12O10]∞ et (b) des rubans de type [Nb4O18]∞.

**Fig. 4.**
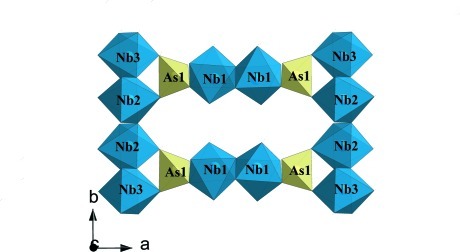
: Vue en perspective, selon c, montrant la jonction entre les rubans et les chaînes ondulèes.

**Fig. 5.**
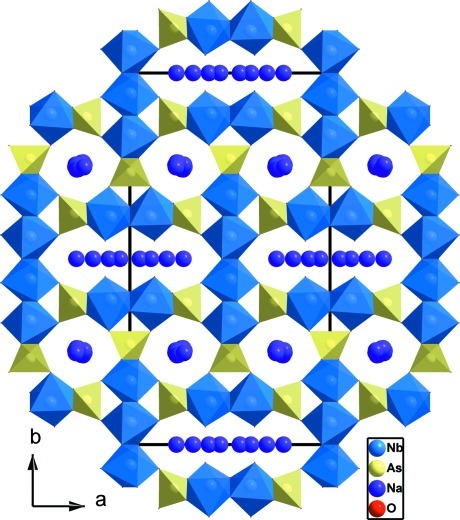
: Projection de la structure de Na3Nb4As3O19 selon c, montrant les canaux où se situent les cations.

## supplementary crystallographic information

### Comment

Dans le cadre de la synthèse des matériaux à charpentes ouvertes, nous avons poursuivi l'exploitation des systèmes A—Nb—As—O (A = alcalin, argent) dans lesquels nous avons précédemment caractérisé les phases suivantes: K_3_NbAs_2_O_9_ (Zid *et al.*, 1989), Ag_3_Nb_3_As_2_O_14_ (Ben Amor & Zid, 2006), Na_3_NbAs_2_O_9_ (Hizaoui *et al.*, 1999*b*), K_2_Nb_2_As_2_O_11_ (Zid *et al.*, 1988), KNb_4_AsO_13_ (Haddad *et al.*, 1988) et K_0.12_Na_0.58_Ag_0.30_Nb_4_AsO_13_ (Chérif *et al.*, 2011).

La structure du composé Na_3_Nb_4_As_3_O_19_ peut être décrite à partir d'octaèdres NbO_6_ et de tétraèdres As1O_4_ partageant leurs sommets. Au sein de la structure (Fig. 1), ces polyèdres forment des groupements classiques cycliques Nb_2_AsO_13_ (Fig. 2 (*a*)) de type *M*_2_XO_13_ (*M* = Mo, Nb et *X* = As, P) similaires à ceux rencontrés dans Ag_3_Nb_3_As_2_O_14_ (Ben Amor & Zid, 2006), LiMo_2_O_3_(PO_4_)_2_ (Ledain *et al.*, 1996) et RbNb_2_PO_8_ (Leclaire *et al.*, 1994) et des unités originales Nb_2_As_2_O_17_ (Fig. 2 (*b*)). Dans la charpente, les octaèdres Nb1O_6_ sont liés par les sommets et forment, selon c, des chaînes ondulées de type [Nb1_2_O_10_]_∞_ (Fig. 3(*a*)) comme celles rencontrées dans NbOPO_4_ (Amos & Sleight, 2001). Les octaèdres Nb2O_6_ et Nb3O_6_ se connectent par les sommets pour former des rubans de type [Nb_4_O_18_]_∞_ (Fig. 3 (*b*)). Ces derniers et les chaînes ondulées [Nb1_2_O_10_]_∞_ sont liés par les sommets par l'intermédiaire de tétraèdres AsO_4_ (Fig. 4). Il en résulte une charpente tridimensionnelle possédant deux types de canaux, disposés parallélement à la direction [001], respectivement de sections très allongées larges et hexagonales où résident les cations Na^+^ (Fig. 5). Les atomes d'arsenic, de niobium et de sodium forment respectivement avec les atomes d'oxygène des liaisons As—O, Nb—O et Na—O conformes à celles rencontrées dans la littérature (Hizaoui *et al.*, 1999*a*; Harrison *et al.*, 1994; Ben Amor & Zid, 2005). Au sein de l'octaèdre Nb(1)O_6_, on *rel*ève une liaison courte caractéristique d'un groupement niobyl (*d*(Nb—O) = 1,762 (5) Å). Le calcul des différentes valences des liaisons (BVS), utilisant la formule empirique de Brown (Brown & Altermatt, 1985), conduit aux valeurs des charges des ions: Nb1(4,92), Nb2(4,98), Nb3(5,14), As1(4,98), As2(5,03), Na1(0,98), Na2(1,04), Na3(1,07), Na4(1,12), Na5(0,79), Na6(0,79), Na7(0,77) et Na8(0,77), en accord avec les degrés d'oxydation attendus. La comparaison de la structure avec celles rencontrées dans la littérature et renfermant les mêmes types de groupements: *M*_2_XO_13_ et *M*_2_*X*_2_O_17_ (*M* = métal de transition, *X* = As, P) révèle une filiation structurale avec les matériaux à charpentes: unidimensionnelle Na_3_SbO(PO_4_)_2_ (Guyomard *et al.*, 1991), bidimensionnelle CaNb_2_P_2_O_11_ (Serra & Hwu, 1992) et tridimensionnelles Ag_3_Nb_3_As_2_O_14_ (Ben Amor & Zid, 2006), LiMo_2_O_3_(PO_4_)_2_ (Ledain *et al.*, 1996) et RbNb_2_PO_8_ (Leclaire *et al.*, 1994). En effet dans cette filiation, ces unités se regroupent, selon les trois directions de l'espace, par établissement de ponts mixtes M—O—*X* pour conduire à diffèrentes structures tridimensionnelles (three-dimensional). Une disposition particulière des groupements: *M*_2_XO_13_ et *M*_2_*X*_2_O_17_ (*M* = Nb et *X* = As) prévoie la formation de deux types de ponts Nb—O—Nb et Nb—O—As et aboutit à une charpente tridimensionnelle (three-dimensional) similaire à celle rencontrée dans notre matériau Na_3_Nb_4_As_3_O_19_. *L*'occupation partielle des sites par Na^+^ dans la stucture du composé Na_3_Nb_4_As_3_O_19_ pourrait conférer à ce matériau des propriétés de conduction ionique (Masquelier *et al.*, 1995; Daidouh *et al.*, 1997, 1998, 1999; Sanz *et al.*, 1999, 2001; Baies *et al.*, 2006). Le composé Na_3_Nb_4_As_3_O_19_, appartenant à une classe non-centrosymétrique (groupe d'espace: *C*222_1_) pourrait présenter des propriétés optiques non linéaires (Ravez *et al.*, 1972, 1974). La présence de groupements niobyl dans la structure laisse prévoir également des propriétés de luminescence (Torardi *et al.*, 1985; Krol *et al.*, 1980; Blasse *et al.*, 1992).

### Experimental

Les cristaux de Na_3_Nb_4_As_3_O_19_ ont été obtenus à partir des réactifs: Nb_2_O_5_ (Fluka, 72520), NH_4_H_2_AsO_4_ (préparé au laboratoire, ASTM 01–775) et Na_2_CO_3_ (Prolabo, 27766) pris dans les proportions Na:Nb:As=5:3:4. Le mélange, finement broyé, a été mis dans un creuset en porcelaine, placé dans un four puis préchauffé à l'air à 523 K pendant 24 heures en vue d'éliminer les composés volatils. Il est ensuite porté à une température proche de sa fusion, 1123 K. Le mélange est maintenu à cette température pendant une semaine pour favoriser la germination et la croissance des cristaux puis il subit en premier lieu un refroidissement lent (5°/jour) jusqu'à 1073 K puis un second rapide (50°/h) jusqu'à la température ambiante. Des cristaux incolores ont été séparés du flux par de l'eau bouillante. Une analyse qualitative au M.E.B de type FEI Quanta 200 d'un cristal choisi, confirme la présence des différents éléments chimiques attendus notamment: Nb, As, Na et l'oxygène.

### Refinement

Les Na^+^ ont été localisés par Fourier-différence sur 1 site Na1 et sur 2 sites éclatés respectivement en 3 sites (Na2, Na3 et Na4) et 4 sites (Na5, Na6, Na7 et Na8). Les distances entre ces sites éclatés sont: Na2—Na4 = 0,761; Na3—Na4 = 0,979; Na5—Na8 = 0,725; Na5—Na6 = 0,759; Na6—Na7 = 0,631 et Na7—Na8 = 0,606 Å. Les taux d'occupation des sites Na^+^ ont été affinés et constraints de façon à assurer l'électroneutralité en utilisant l'option SUMP du programme *SHELX* (Sheldrick, 2008). *L*'affinement des paramètres de déplacement atomique conduit à des ellipsoïdes bien définis. Les densités électroniques résiduelles maximale et minimale observées sur la Fourier-différence finale sont situées respectivement à 1,07 Å de O9 et à 0,97 Å de Nb2.

### Crystal data

**Table:**

Na_3_Nb_4_As_3_O_19_	*F*(000) = 1792
*M**_r_* = 969.37	*D*_x_ = 4.023 Mg m^−^^3^
Orthorhombic, *C*222_1_	Mo *K*α radiation, λ = 0.71073 Å
Hall symbol: C 2c 2	Cell parameters from 25 reflections
*a* = 13.014 (2) Å	θ = 10–15°
*b* = 24.170 (3) Å	µ = 9.13 mm^−^^1^
*c* = 5.0880 (9) Å	*T* = 298 K
*V* = 1600.4 (3) Å^3^	Prism, colourless
*Z* = 4	0.35 × 0.25 × 0.16 mm

### Data collection

**Table:**

Enraf–Nonius CAD-4 diffractometer	1516 reflections with *I* > 2σ(*I*)
Radiation source: fine-focus sealed tube	*R*_int_ = 0.041
Graphite monochromator	θ_max_ = 27.0°, θ_min_ = 3.0°
ω/2θ scans	*h* = −1→16
Absorption correction: ψ scan (North *et al.*, 1968)	*k* = −1→30
*T*_min_ = 0.083, *T*_max_ = 0.230	*l* = −6→6
2116 measured reflections	2 standard reflections every 120 min
1757 independent reflections	intensity decay: 1%

### Refinement

**Table:**

Refinement on *F*^2^	Primary atom site location: structure-invariant direct methods
Least-squares matrix: full	Secondary atom site location: difference Fourier map
*R*[*F*^2^ > 2σ(*F*^2^)] = 0.029	*w* = 1/[σ^2^(*F*_o_^2^) + (0.0368*P*)^2^] where *P* = (*F*_o_^2^ + 2*F*_c_^2^)/3
*wR*(*F*^2^) = 0.076	(Δ/σ)_max_ = 0.001
*S* = 1.03	Δρ_max_ = 0.75 e Å^−^^3^
1757 reflections	Δρ_min_ = −0.91 e Å^−^^3^
159 parameters	Absolute structure: Flack (1983), 718 Friedel pairs
2 restraints	Flack parameter: 0.019 (16)

### Special details

**Table:**

Geometry. All e.s.d.'s (except the e.s.d. in the dihedral angle between two l.s. planes) are estimated using the full covariance matrix. The cell e.s.d.'s are taken into account individually in the estimation of e.s.d.'s in distances, angles and torsion angles; correlations between e.s.d.'s in cell parameters are only used when they are defined by crystal symmetry. An approximate (isotropic) treatment of cell e.s.d.'s is used for estimating e.s.d.'s involving l.s. planes.
Refinement. Refinement of *F*^2^ against ALL reflections. The weighted *R*-factor *wR* and goodness of fit *S* are based on *F*^2^, conventional *R*-factors *R* are based on *F*, with *F* set to zero for negative *F*^2^. The threshold expression of *F*^2^ > σ(*F*^2^) is used only for calculating *R*-factors(gt) *etc*. and is not relevant to the choice of reflections for refinement. *R*-factors based on *F*^2^ are statistically about twice as large as those based on *F*, and *R*- factors based on ALL data will be even larger.

### Fractional atomic coordinates and isotropic or equivalent isotropic displacement parameters (Å^2^)

**Table:**

	*x*	*y*	*z*	*U*_iso_*/*U*_eq_	Occ. (<1)
Nb1	0.09989 (4)	0.61182 (2)	0.98822 (12)	0.00837 (14)	
Nb2	0.5000	0.55734 (4)	0.7500	0.0191 (2)	
Nb3	0.5000	0.66841 (3)	0.2500	0.0147 (2)	
As1	0.28249 (5)	0.61930 (3)	0.51616 (17)	0.00926 (16)	
As2	0.0000	0.73103 (4)	0.2500	0.0116 (2)	
Na1	0.2677 (4)	0.5000	0.0000	0.066 (3)	0.937 (16)
Na2	0.0494 (12)	0.5000	0.5000	0.044 (4)	0.68 (3)
Na3	0.184 (4)	0.5000	0.5000	0.027 (18)*	0.092 (18)
Na4	0.105 (7)	0.5000	0.5000	0.029 (17)*	0.11 (3)
Na5	0.771 (3)	0.7465 (10)	0.753 (13)	0.014 (11)*	0.12 (2)
Na6	0.742 (4)	0.7414 (15)	0.629 (18)	0.022 (14)*	0.11 (3)
Na7	0.7659 (13)	0.7594 (7)	0.006 (11)	0.010 (5)*	0.191 (16)
Na8	0.7726 (19)	0.7525 (11)	0.892 (12)	0.018 (8)*	0.17 (2)
O1	0.3557 (4)	0.6722 (2)	0.3978 (9)	0.0126 (11)	
O2	0.0000	0.4110 (3)	0.7500	0.0139 (14)	
O3	0.2178 (4)	0.64941 (19)	0.7635 (11)	0.0148 (10)	
O4	0.1043 (4)	0.6949 (2)	0.1612 (10)	0.0151 (10)	
O5	0.2132 (4)	0.5923 (2)	0.2719 (12)	0.0156 (11)	
O6	0.6461 (4)	0.56446 (17)	0.8803 (9)	0.0096 (10)	
O7	0.4590 (5)	0.5000	0.0000	0.0149 (14)	
O8	0.1235 (4)	0.5478 (2)	0.8340 (10)	0.0151 (10)	
O9	−0.0435 (4)	0.7711 (2)	0.0017 (15)	0.0264 (14)	
O10	0.0000	0.6408 (3)	0.7500	0.0150 (15)	
O11	0.4634 (4)	0.61482 (19)	0.0053 (14)	0.0234 (13)	

### Atomic displacement parameters (Å^2^)

**Table:**

	*U*^11^	*U*^22^	*U*^33^	*U*^12^	*U*^13^	*U*^23^
Nb1	0.0069 (3)	0.0093 (3)	0.0089 (3)	−0.00043 (19)	0.0005 (4)	−0.0009 (2)
Nb2	0.0102 (4)	0.0107 (4)	0.0364 (6)	0.000	−0.0103 (5)	0.000
Nb3	0.0139 (4)	0.0088 (4)	0.0212 (4)	0.000	0.0098 (4)	0.000
As1	0.0060 (3)	0.0116 (3)	0.0101 (4)	0.0003 (2)	0.0005 (3)	0.0015 (3)
As2	0.0091 (5)	0.0072 (4)	0.0186 (5)	0.000	−0.0002 (5)	0.000
Na1	0.012 (3)	0.036 (4)	0.150 (8)	0.000	0.000	0.036 (6)
Na2	0.045 (8)	0.024 (4)	0.063 (7)	0.000	0.000	−0.026 (5)
O1	0.007 (2)	0.015 (3)	0.015 (3)	0.000 (2)	−0.0004 (19)	0.004 (2)
O2	0.010 (3)	0.020 (3)	0.012 (3)	0.000	−0.006 (3)	0.000
O3	0.013 (2)	0.014 (2)	0.017 (3)	−0.0008 (19)	0.004 (3)	−0.003 (2)
O4	0.008 (2)	0.015 (2)	0.022 (3)	0.000 (2)	−0.001 (2)	−0.005 (2)
O5	0.010 (2)	0.018 (2)	0.019 (3)	−0.002 (2)	−0.007 (2)	0.000 (2)
O6	0.011 (2)	0.002 (2)	0.016 (3)	−0.0010 (19)	−0.005 (2)	0.0001 (18)
O7	0.012 (3)	0.012 (3)	0.020 (4)	0.000	0.000	0.009 (4)
O8	0.016 (2)	0.010 (2)	0.020 (2)	0.002 (2)	0.001 (2)	−0.006 (2)
O9	0.016 (3)	0.022 (3)	0.041 (4)	−0.003 (2)	−0.001 (3)	0.025 (3)
O10	0.014 (3)	0.016 (3)	0.015 (4)	0.000	−0.005 (4)	0.000
O11	0.011 (2)	0.023 (3)	0.037 (3)	−0.0011 (18)	−0.004 (3)	−0.016 (4)

### Geometric parameters (Å, º)

**Table:**

Nb1—O8	1.762 (5)	Na2—O8^x^	2.270 (9)
Nb1—O10	1.910 (3)	Na2—O2^xiii^	2.580 (7)
Nb1—O2^i^	1.941 (3)	Na2—O2	2.580 (7)
Nb1—O5^ii^	2.117 (6)	Na2—O8^xiii^	2.667 (14)
Nb1—O3	2.119 (5)	Na2—O8^xiv^	2.667 (14)
Nb1—O4^ii^	2.194 (5)	Na3—O8^x^	2.202 (19)
Nb2—O7^iii^	1.955 (2)	Na3—O8	2.202 (19)
Nb2—O7^ii^	1.955 (2)	Na3—O5	2.542 (9)
Nb2—O11^ii^	1.961 (6)	Na3—O5^x^	2.542 (9)
Nb2—O11^iv^	1.961 (6)	Na3—O6^v^	2.77 (4)
Nb2—O6^v^	2.021 (5)	Na3—O6^xii^	2.77 (4)
Nb2—O6	2.021 (5)	Na4—O8	2.069 (11)
Nb3—O11	1.859 (6)	Na4—O8^x^	2.069 (11)
Nb3—O11^iv^	1.859 (6)	Na4—O2^xiii^	2.85 (4)
Nb3—O9^vi^	2.024 (6)	Na4—O2	2.85 (4)
Nb3—O9^vii^	2.024 (6)	Na4—O5	2.88 (4)
Nb3—O1	2.025 (5)	Na4—O5^x^	2.88 (4)
Nb3—O1^iv^	2.025 (5)	Na5—O3^v^	2.35 (3)
As1—O5	1.669 (6)	Na5—O1^xv^	2.38 (2)
As1—O3	1.680 (6)	Na5—O4^xv^	2.62 (2)
As1—O6^v^	1.702 (4)	Na5—O9^xvi^	2.80 (5)
As1—O1	1.705 (5)	Na6—O3^v^	2.35 (4)
As2—O4^viii^	1.676 (5)	Na6—O1^xv^	2.56 (4)
As2—O4	1.676 (5)	Na6—O4^xv^	2.59 (4)
As2—O9^viii^	1.690 (6)	Na6—O9^vi^	2.68 (6)
As2—O9	1.690 (6)	Na7—O9^xvii^	2.496 (17)
Na1—O8^ix^	2.360 (7)	Na7—O4^vii^	2.523 (18)
Na1—O8^x^	2.360 (7)	Na7—O3^xv^	2.57 (4)
Na1—O7	2.489 (8)	Na7—O1^iv^	2.681 (17)
Na1—O5	2.718 (6)	Na8—O9^xvi^	2.50 (3)
Na1—O5^xi^	2.718 (6)	Na8—O4^xv^	2.55 (2)
Na1—O6^iv^	2.726 (6)	Na8—O1^xv^	2.58 (3)
Na1—O6^xii^	2.726 (6)	Na8—O3^v^	2.62 (4)
Na2—O8	2.270 (9)		
			
O8—Nb1—O10	99.1 (2)	O6^v^—Nb2—O6	170.2 (2)
O8—Nb1—O2^i^	99.9 (3)	O11—Nb3—O11^iv^	91.6 (4)
O10—Nb1—O2^i^	94.80 (12)	O11—Nb3—O9^vi^	177.1 (3)
O8—Nb1—O5^ii^	89.2 (2)	O11^iv^—Nb3—O9^vi^	90.5 (3)
O10—Nb1—O5^ii^	171.3 (2)	O11—Nb3—O9^vii^	90.5 (3)
O2^i^—Nb1—O5^ii^	86.28 (18)	O11^iv^—Nb3—O9^vii^	177.1 (3)
O8—Nb1—O3	90.6 (2)	O9^vi^—Nb3—O9^vii^	87.5 (4)
O10—Nb1—O3	89.64 (18)	O11—Nb3—O1	92.5 (2)
O2^i^—Nb1—O3	167.8 (2)	O11^iv^—Nb3—O1	91.2 (2)
O5^ii^—Nb1—O3	87.7 (2)	O9^vi^—Nb3—O1	89.5 (2)
O8—Nb1—O4^ii^	168.0 (2)	O9^vii^—Nb3—O1	86.7 (2)
O10—Nb1—O4^ii^	86.4 (2)	O11—Nb3—O1^iv^	91.2 (2)
O2^i^—Nb1—O4^ii^	90.2 (2)	O11^iv^—Nb3—O1^iv^	92.5 (2)
O5^ii^—Nb1—O4^ii^	85.0 (2)	O9^vi^—Nb3—O1^iv^	86.7 (2)
O3—Nb1—O4^ii^	78.75 (19)	O9^vii^—Nb3—O1^iv^	89.5 (2)
O7^iii^—Nb2—O7^ii^	89.72 (14)	O1—Nb3—O1^iv^	174.8 (3)
O7^iii^—Nb2—O11^ii^	178.2 (2)	O5—As1—O3	117.2 (3)
O7^ii^—Nb2—O11^ii^	90.3 (2)	O5—As1—O6^v^	102.7 (2)
O7^iii^—Nb2—O11^iv^	90.3 (2)	O3—As1—O6^v^	112.3 (3)
O7^ii^—Nb2—O11^iv^	178.2 (2)	O5—As1—O1	109.4 (3)
O11^ii^—Nb2—O11^iv^	89.8 (4)	O3—As1—O1	102.7 (2)
O7^iii^—Nb2—O6^v^	95.9 (2)	O6^v^—As1—O1	112.8 (2)
O7^ii^—Nb2—O6^v^	91.0 (2)	O4^viii^—As2—O4	117.2 (3)
O11^ii^—Nb2—O6^v^	85.9 (2)	O4^viii^—As2—O9^viii^	111.6 (3)
O11^iv^—Nb2—O6^v^	87.2 (2)	O4—As2—O9^viii^	103.2 (2)
O7^iii^—Nb2—O6	91.0 (2)	O4^viii^—As2—O9	103.2 (2)
O7^ii^—Nb2—O6	95.9 (2)	O4—As2—O9	111.6 (3)
O11^ii^—Nb2—O6	87.2 (2)	O9^viii^—As2—O9	110.0 (4)
O11^iv^—Nb2—O6	85.9 (2)		

Symmetry codes: (i) −*x*, −*y*+1, *z*+1/2; (ii) *x*, *y*, *z*+1; (iii) −*x*+1, −*y*+1, *z*+1/2; (iv) −*x*+1, *y*, −*z*+1/2; (v) −*x*+1, *y*, −*z*+3/2; (vi) −*x*+1/2, −*y*+3/2, *z*+1/2; (vii) *x*+1/2, −*y*+3/2, −*z*; (viii) −*x*, *y*, −*z*+1/2; (ix) *x*, *y*, *z*−1; (x) *x*, −*y*+1, −*z*+1; (xi) *x*, −*y*+1, −*z*; (xii) −*x*+1, −*y*+1, *z*−1/2; (xiii) −*x*, −*y*+1, *z*−1/2; (xiv) −*x*, *y*, −*z*+3/2; (xv) *x*+1/2, −*y*+3/2, −*z*+1; (xvi) *x*+1, *y*, *z*+1; (xvii) *x*+1, *y*, *z*.
